# Measuring brain atrophy with a generalized formulation of the boundary shift integral^☆^

**DOI:** 10.1016/j.neurobiolaging.2014.04.035

**Published:** 2015-01

**Authors:** Ferran Prados, Manuel Jorge Cardoso, Kelvin K. Leung, David M. Cash, Marc Modat, Nick C. Fox, Claudia A.M. Wheeler-Kingshott, Sebastien Ourselin

**Affiliations:** a Centre for Medical Image Computing (CMIC), Department of Medical Physics and Bioengineering, University College London, London, UK; b NMR Research Unit, Queen Square MS Centre, Department of Neuroinflammation, UCL Institute of Neurology, London, UK; c Dementia Research Centre, Department of Neurodegenerative Disease, UCL Institute of Neurology, London, UK

**Keywords:** boundary shift integral, Alzheimer's disease, Clinical trials, MRI, Biomarker

## Abstract

Brain atrophy measured using structural magnetic resonance imaging (MRI) has been widely used as an imaging biomarker for disease diagnosis and tracking of pathologic progression in neurodegenerative diseases. In this work, we present a generalized and extended formulation of the boundary shift integral (gBSI) using probabilistic segmentations to estimate anatomic changes between 2 time points. This method adaptively estimates a non-binary exclusive OR region of interest from probabilistic brain segmentations of the baseline and repeat scans to better localize and capture the brain atrophy. We evaluate the proposed method by comparing the sample size requirements for a hypothetical clinical trial of Alzheimer's disease to that needed for the current implementation of BSI as well as a fuzzy implementation of BSI. The gBSI method results in a modest but reduced sample size, providing increased sensitivity to disease changes through the use of the probabilistic exclusive OR region.

## 1 Introduction

Imaging biomarkers have become a key tool for early detection, differential diagnosis, and disease progression in neurodegenerative diseases in the last decade (Gustaw-Rothenberg et al., 2010). Using these biomarkers as outcome measures in trials would also have the potential to show a disease modifying effect on fewer subjects than standard cognitive tests, with proper enrichment strategies making these useful for predementia trials. (Grill et al., 2013; Schott et al., 2010).

Rates of whole brain and hippocampal atrophy from longitudinal magnetic resonane imaging (MRI) scans can aid in disease diagnosis and tracking of pathologic progression in neurodegenerative diseases and are increasingly used as outcome measures in trials of potentially disease-modifying therapies (Anderson et al., 2006; Frisoni et al., 2010; Holland et al., 2012; Sharma et al., 2010; Sluimer et al., 2010). Popular methods for brain atrophy measurement in longitudinal studies include Boundary Shift Integral (BSI) (Freeborough and Fox, 1997; Leung et al., 2010b, 2012), Structural Image Evaluation, using Normalization, of Atrophy (SIENA) (Smith et al., 2001), Quantitative Anatomical Regional Change (QUARC) (Holland and Dale, 2011), Tensor-Based Morphometry (TBM) (Hua et al., 2013), and FreeSurfer-longitudinal (FS) (Reuter et al., 2012). BSI and SIENA both use linear registration to align the baseline and repeat images and then track the shift of the brain boundary location, whereas QUARC and TBM both use nonlinear registrations to map between the baseline and repeat images and then measure volume change through analysis of the resulting deformation fields. FS is based on performing independent tissue segmentation at each time point and build subject-specific average from the time points. These analyses can be limited to specific ROIs, such as the entorhinal cortex or the hippocampus, to better localize where atrophy is occurring.

BSI has been shown to provide accurate measurements of brain atrophy that are sensitive biomarkers of disease progression (Leung et al., 2012). The pipeline consists of several processing steps, including intensity normalization, segmentation, registration, and differential bias correction (Leung et al., 2010b). A key step in the BSI pipeline is the region extraction process. It is essential that the boundary defining the region of interest be accurate, defining the interface between tissue and cerebrospinal fluid (CSF), correctly detecting sulcal and ventricular boundaries, to produce an accurate and robust measurement of atrophy. Whether automatically segmented or manually delineated by trained experts, there still will be some partial volume effects or segmentation errors that remain. Thus, a boundary shift region is created from the extracted regions of baseline and repeat scans by performing an exclusive OR (XOR) operation on the dilated union and eroded intersection regions of the baseline and repeat binary masks. However, this operation may still cause non-brain tissues to be included (e.g., dura), which may introduce noise, and thus, reduce sensitivity to the measurement. The consistency and test-retest reproducibility of BSI has been demonstrated recently in Leung et al. (2012).

Ledig et al. (2012) proposed to modify the BSI method by using probabilistic segmentations of the brain and other regions of interest (the method is referred as “pBSI”). The probabilistic masks from the baseline and repeat scans were combined through a fuzzy union and intersection and then binarized using parameterized thresholds. The boundary shift region was then created using the dilated union region and the eroded intersection region, which may still cause non-brain tissues to be included. Then, the XOR region was weighted according to the probability that it contained brain tissue. Finally, the BSI integral was calculated using the weighted XOR region.

The pBSI method (Ledig et al., 2012) is based on standard BSI (Freeborough and Fox, 1997), where BSI is calculated with a manual and fixed intensity window rather than performing tissue-specific intensity normalization and parameter selection done by “KN-BSI” (Leung et al., 2010b). Moreover, measuring hippocampal atrophy means that we have to apply a double intensity window to capture boundary shift at both the hippocampal GM-CSF border and the hippocampal GM-WM border (Hobbs et al., 2009). Finally, according to Lindley (1987), a probabilistic formulation is a more sensible description of uncertainty than the fuzzy framework used in Ledig et al. (2012) and should be ideal for all problems involving uncertainty.

In this work, we propose a generalized formulation of the BSI, which incorporates probabilistic spatial information, because as Manjón et al. (2010) demonstrated, using spatial information in combination with an appropriate tissue parameter estimation improves the tissue volume estimation. The algorithm adaptively estimates a non-binary XOR region of interest from probabilistic brain segmentations of the baseline and repeat scans using probabilistic logic operations to better localize and capture the brain atrophy. The proposed method uses the probabilistic segmentations obtained from a multiatlas propagation and label fusion algorithm (Cardoso et al., 2013) to adaptively select a spatial window. The aim of the proposed framework is to increase the sensitivity to disease-related change. We evaluated the proposed method by comparing atrophy rates and sample sizes to the current implementation of our KN-BSI method (Leung et al., 2010b) and pBSI method (Ledig et al., 2012).

## 2 Methods

### 2.1 MRI data

Data used in the preparation of this article were obtained from the ADNI database (www.loni.ucla.edu/ADNI), which was launched in 2003. The primary goal of ADNI has been to test whether serial MRI, positron emission tomography, other biological markers, and clinical and neuropsychological assessment can be combined to measure the progression of mild cognitive impairment and early Alzheimer's disease (AD). Determination of sensitive and specific markers of very early AD progression is intended to aid researchers and clinicians to develop new treatments and monitor their effectiveness, as well as lessen the time and cost of clinical trials.

The Principal Investigator of this initiative is Michael W. Weiner, MD, VA Medical Center and University of California, San Francisco. ADNI is the result of efforts of many coinvestigators from a broad range of academic institutions and private corporations, and subjects have been recruited from over 50 sites across the United States and Canada. The initial goal of ADNI was to recruit 800 adults, aged 55–90 years to participate in the research (approximately 200 cognitively normal older individuals, 400 people with mild cognitive impairment, and 200 people with early AD). For up-to-date information, see http://www.adni-info.org.

In this work, we used baseline and 12 months follow-up scans of 328 subjects at 1.5 T (195 controls and 155 AD) and 63 subjects at 3T (39 controls and 24 AD), which represents the ADNI-1 subjects available for standard analysis data sets who had T1-weighted MRI scans at baseline, 6 months, and 12 months (Wyman et al., 2013).

All images downloaded from the ADNI database had already been preprocessed through the standard pipeline. This pipeline includes N3 correction for image inhomogeneity (Sled et al., 1998), B1 nonuniformity correction (Narayana et al., 1988), GradWarp correction for geometric distortion (Jovicich et al., 2006), and phantom-based scaling correction (Gunter et al., 2006)—the geometric phantom scan having been acquired with each patient scan.

### 2.2 Template library

The template library used in this work consisted of the 682 1.5 T MRI images from the baseline scans of ADNI. For each image in the template library, we had associated manual segmentations of the brain. We also had manual segmentations of 55 left and 55 right hippocampal, which were flipped along the left-right as in Leung et al. (2010b) to increase the template library to 110 samples.

Because both the brain template library and the image data are from ADNI, a leave-one-out cross-validation approach is used, that is, the target image is excluded from the template library.

### 2.3 Pipeline overview

**Fig. 1:**
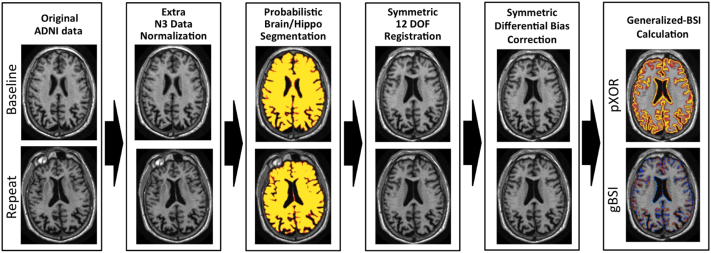
Diagram representing the gBSI different processing steps for atrophy estimation. Abbreviations: ADNI, Alzheimer's Disease Neuroimaging Initiative; BSI, boundary shift integral; DOF, degrees of freedom; gBSI, generalized boundary shift integral.

**Fig. 2:**
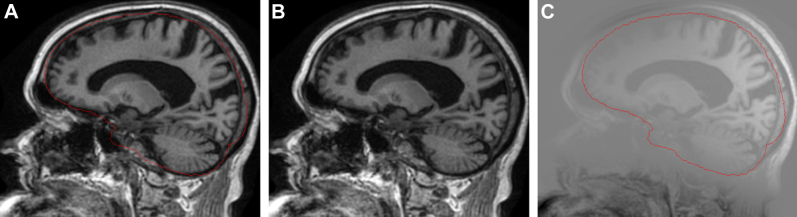
Extra N3 correction. (A) Initial scan with the template-12-dof mask overlaid. (B) Corrected scan. (C) Subtraction between initial scan and corrected scan.

An overview of the whole pipeline is shown in Fig. 1. An extra preprocessing step for intensity inhomogeneity correction was applied to the ADNI scans using a robust version of the N3 algorithm, as proposed in Boyes et al. (2008), see Fig. 2. The preprocessed scans were independently segmented using a segmentation propagation and fusion method, which provided probabilistic masks for each image. The next step was a symmetric and inverse-consistent registration to the middle space of the 2 time-point images using 12 degrees of freedom (DOF) registration (Modat et al., 2014). A symmetric differential bias correction (DBC) was then applied to both registered images to reduce the residual bias field between them. Finally, the atrophy was calculated using the proposed generalized BSI method, denoted as gBSI.

#### 2.3.1 Multiatlas similarity segmentation

Probabilistic masks were obtained using a multi-atlas segmentation propagation and fusion technique called STEPS (Cardoso et al., 2013). This segmentation process is divided in 2 stages: segmentation propagation and fusion. Starting from a template library with associated manual segmentations, all the templates (excluding the image under analysis) are first registered to the target image. The normalized cross correlation (NCC) is then estimated between each deformed template and the target image, quantifying the similarity between 2 images. For the whole brain (hippocampus), the 30 (15) most similar deformed templates according to the NCC are fused into a consensus segmentation according to the locally NCC between the registered template images and the target image. A consensus probabilistic brain and hippocampal segmentation is obtained using the STEPS algorithm, as implemented in NiftySeg. The probabilistic nature of the consensus segmentation implicitly encodes segmentation uncertainty, improving sulcal delineation and tissue boundary localization.

#### 2.3.2 Symmetric and inverse-consistent registration

The use of a symmetric and inverse-consistent registration ensures that the BSI findings are unbiased toward the directionality of the registration process. Using the obtained transformations, all input images are resampled to a middle space (Reuter et al., 2010; Smith et al., 2002). It ensures that all images are treated similarly as they all receive the same degree of interpolation-related blurring. The symmetric full affine approach (Modat et al., 2014), 12 DOFs, that we used, is based on the asymmetric block-matching approach initially described by Ourselin et al. (2001). The forward and backward transformations are optimized concurrently in an inverse-consistent manner. The implementation is freely available from the NiftyReg package.

Similarly to previous work by Leung et al. (2012), all registrations were performed by considering 8-voxel dilated brain regions of interest. Note that contrary to the previous version of BSI (Freeborough and Fox, 1997; Leung et al., 2010b), which used 9 DOF (includes translation, rotation, and scale parameters), we use 12 DOF instead of the 9 DOF because 9 DOF registration is inherently asymmetric (Leung et al., 2012). This asymmetry could then introduce a bias in the atrophy estimates. If either image can be scaled anisotropically along their own axes, and the images are acquired such that these axes need to be rotated to align anatomy, then the separate scalings together with the rotation between the pairs of axes effectively allow skews. More formally, 12 DOF transformations form a matrix Lie group with an associated semi-Riemannian manifold so their inverses and compositions are also 12 DOF; this is not generally true of 9 DOF transformations, whose inverses or compositions are only guaranteed to be within the broader 12 DOF group. Thus, we parameterize the 12 DOF transformation as 3 translations, 3 rotations in Euler angles, 3 scaling factors, and 3 skew factors, and the full matrix is optimized directly.

#### 2.3.3 Symmetric differential bias correction

Although the data has been previously corrected for intensity inhomogeneity using N3 Boyes et al. (2008), a symmetric DBC is also applied to the registered baseline and repeat images. The DBC is used to correct the residual intensity inhomogeneity-derived differences between the baseline and the repeat images. A DBC kernel with a radius of 5 was used for all experiments (Lewis and Fox, 2004).

### 2.4 Generalized boundary shift integral

The BSI can be described by 4 different steps: (1) image are normalized according to the average tissue intensity; (2) the intensity clipping window is computed; (3) the probabilistic boundary-shift region of interest is obtained; and (4) the BSI integral is finally estimated.

#### 2.4.1 Intensity normalization

DBC-baseline and repeat half-way registered images are intensity normalized using linear regression coefficients (Leung et al., 2010b). These coefficients are obtained from the computation of mean intensities of CSF, GM, WM, and the interior brain region using a *k*-means clustering algorithm. The *k*-means is restricted to a region of interest defined as the 0.5 thresholded and binarized probabilistic brain mask, further dilated by 3 voxels to include some CSF.

#### 2.4.2 Intensity clipping window calculation

The intensity clipping windows [*I*_*low*_, *I*_*high*_] for each image are obtained from:

(1) $$I_{\text{low}}=\text{mean}\left(I_{\text{CSF}}\right)+\text{std}\left(I_{\text{CSF}}\right)$$

and

(2) $$I_{\text{high}}=\text{mean}\left(I_{\text{GM}}\right)-\text{std}\left(I_{\text{GM}}\right)$$

The BSI intensity clipping window is then defined as the average of the intensity windows of the 2 time points.

#### 2.4.3 Probabilistic boundary shift region

Using probabilistic operations, we calculate the exclusive OR region (pXOR) from the half-way resampled consensus region obtained with STEPS. The pXOR is defined as:

(3) $$\text{pXOR}\left(A,B\right)=\left(A\times \bar{B}\right)+\left(\bar{A}\times B\right)-\left(\left(A\times \bar{B}\right)\times \left(\bar{A}\times B\right)\right)$$

where *A* and *B* corresponds to the half-way registered baseline and repeat probabilistic regions, respectively and $\bar{A}$ and $\bar{B}$ to their complement. The pXOR value approaches 1 when the segmentations disagree between the 2 time points, for example, when 1 time point has a very high probability to belong to the ROI, whereas the other time point has a very low probability. After the pXOR calculation each voxel $\left(x,y,z\right)\;\varepsilon \;pXOR\left(R_{t},R_{t+1}\right)$ is weighted by a gain factor *κ*, using the following criterion:

(4) $$R_{\text{pXOR}}=\{\begin{array}{cc} \frac{pXOR\left(x,y,z\right)}{\kappa } & \text{ if }\;\text{pXOR}\left(x,y,z\right)<\kappa \\ 1 & \text{ otherwise}. \end{array}$$

We used the mean of all non-zero voxels of the pXOR region as a *κ* value. The use of the mean, instead of a fixed value, provides an adaptive behavior to our algorithm. The gain factor *κ* acts in a similar way to the dilation and/or erosion operations in the classic BSI, increasing the size of the region of interest. Note that, if we use a *κ* of 1 and if the brain mask is binarised to 0.5, then gBSI will revert to the classic KN-BSI formulation.

**Fig. 3:**
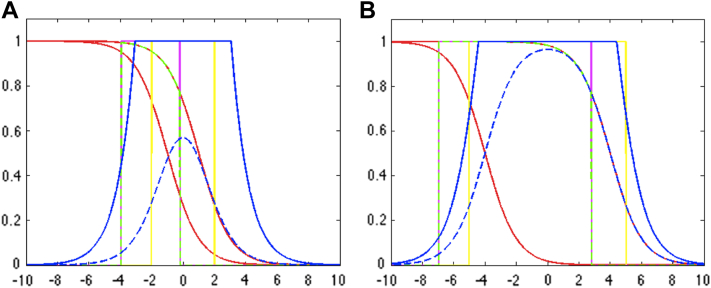
Comparison between binary XOR of the previous BSI (Freeborough and Fox, 1997; Leung et al., 2010b, 2012), fuzzy XOR of pBSI with *γ* ≡ 1 and *γ* ≡ 0.5 (Ledig et al., 2012) and probabilistic weighted XOR of gBSI. X axis represent the tissue displacement along the boundary, Y axis represent segmentation probabilities and red lines represent the probabilistic segmentation of the baseline and repeat images. Different boundary shifts and slope-rates are used to simulate a control (A) and an AD brain (B). The yellow line is the representation of the binary XOR from the classic BSI. This region of interest is produced by thresholding the probability at 0.5 followed by the dilation and/or erosion of the boundaries for XOR estimation. The magenta line is fuzzy XOR of pBSI with *γ* ≡ 1, and the partial overlapped dashed green line is using *γ* ≡ 0.5. Dashed blue line is the estimated pXOR, and blue line corresponds to *R*_*pxOR*_. Abbreviations: AD, Alzheimer's disease; BSI, boundary shift integral; gBSI, generalized boundary shift integral. (For interpretation of the references to color in this Figure, the reader is referred to the web version of this article.)

The differences between the binary XOR and pXOR are shown in Fig. 3. Fig. 3A shows low uncertainty and small shift between 2 masks, reflecting the situation where the boundaries are well defined, that is, for control patients. On the other hand, Fig. 3B represents a high uncertainty configuration, simulating the existence of atrophy and uncertainty between the 2 time points, that is, in AD patients.

**Fig. 4:**
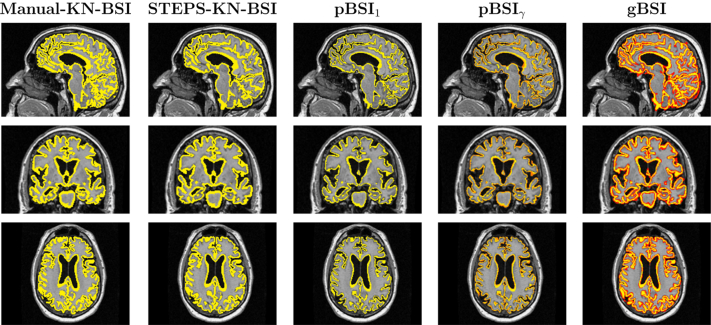
Example of whole brain XOR regions, for manual KN-BSI, STEPS-KN-BSI, pBSI_1_, pBSI_*γ*_, and gBSI, obtained on an AD patient. In yellow binary XOR regions and in a red-yellow scale the XOR pBSI_*γ*_ and gBSI values from 0 to 1. Abbreviations: AD, Alzheimer's disease; BSI, boundary shift integral; gBSI, generalized boundary shift integral. (For interpretation of the references to color in this Figure, the reader is referred to the web version of this article.)

**Fig. 5:**
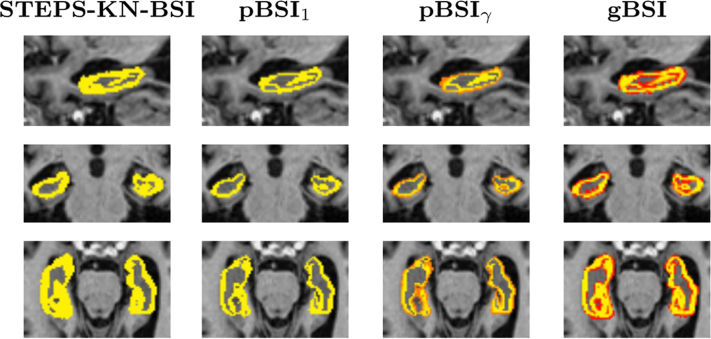
Example of hippocampus XOR regions, for STEPS-KN-BSI, pBSI_1_, pBSI_*γ*_, and gBSI, obtained on an AD patient. In yellow binary XOR regions and in a red-yellow scale the XOR gBSI values from 0 to 1. Abbreviations: AD, Alzheimer's disease; BSI, boundary shift integral; gBSI, generalized boundary shift integral. (For interpretation of the references to color in this Figure, the reader is referred to the web version of this article.)

Fig. 4 and Fig. 5 illustrate the resulting XOR regions for the various implementations of the BSI. The pXOR area (last column) appears quite similar to the conventional KN-BSI XOR region, except that the periphery of the region is weighted to be less than 1. It also appears to be generally more sensitive to the presence of closed sulci than the binary XOR, improving atrophy detection as illustrated by the red regions in these areas. The *κ* gain factor boosts the relevance of voxels surrounding the ROI boundary.

#### 2.4.4 Generalized boundary shift integral

gBSI is calculated for each voxel (*x,y,z*) of the whole volume *V*. The proposed generalized formulation takes into account the differences between clipped image intensities weighted by the edge's membership function *R*_*pxOR*_ (*x,y,z*). Therefore, gBSI is defined as:

(5) $$gBSI=D\sum_{x,y,z\varepsilon V}R_{\text{pXOR}}\left(x,y,z\right)\left(\text{clip}\left(I_{A}\left(x,y,z\right)\right)-\text{clip}\left(I_{B}\left(x,y,z\right)\right)\right)$$

where D is the voxel volume in mm^3^ and *clip* is a function defined as:

(6) $$\text{clip}\left(I\left(x,y,z\right)\right)=\frac{\text{min}\left(\text{max}\left(I\left(x,y,z\right),I_{\text{low}}\right),I_{\text{high}}\right)-I_{low}}{I_{\text{high}}-I_{\text{low}}}\;$$

### 2.5 Parameter choice

There are parameters in each BSI step that could be tuned for an optimal result for a specific data cohort. However, the parameter choices used in this study have been well validated in previous papers and are used in our standard practice pipeline that has been used for thousands of brain scans at our centre. We keep them fixed for all our experiments, Table 1 presents the full parameter list that we have used.

The proposed process provides a fully automated and highly robust methodology for image analysis, without the need for human interaction. Although no failure was observed with the present study, as with any automated method, there might be a possibility of failure for some subjects. Nonetheless, 2 approaches can be used to mitigate these problems: either use the data from subjects that failed in a normal manner, but use robust statistics to detect and remove the influence of outlier data points in population studies or have a staged quality control process with a manual correction of the most sensitive and error prone procedures (e.g., affine registration to the template space).

## 3 Evaluation

**Fig. 6:**
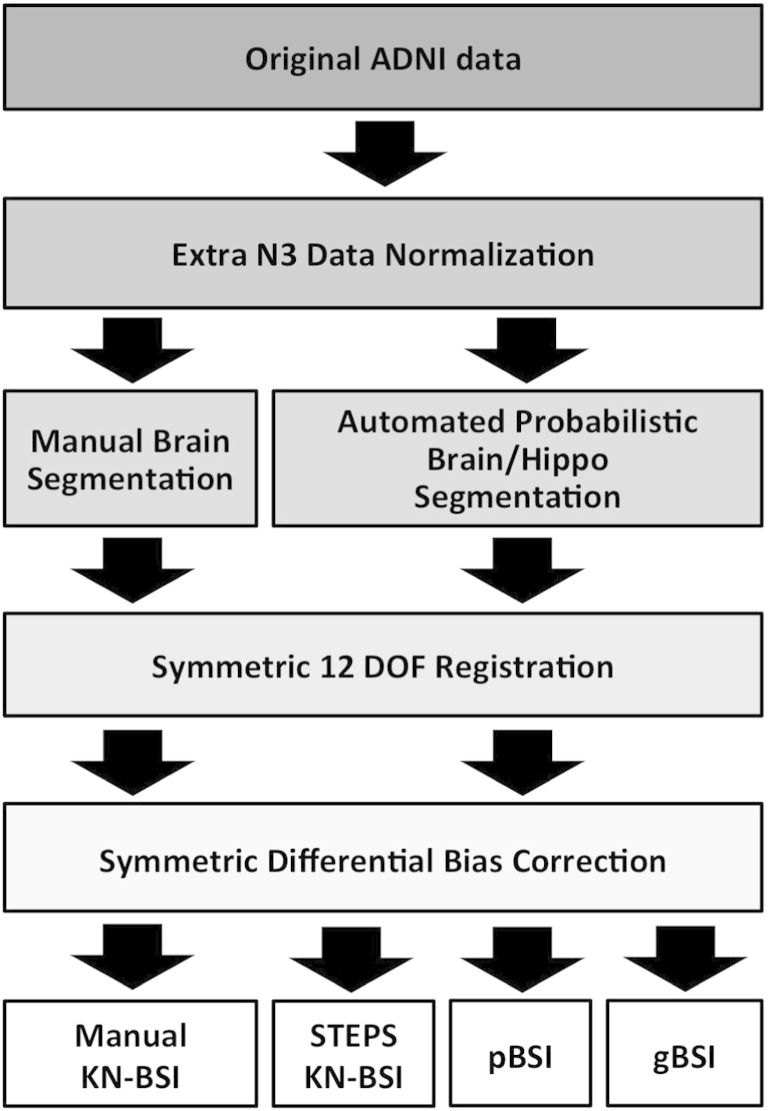
Diagram representing manual-KN-BSI, STEPS-KN-BSI, pBSI, and gBSI processing pipelines. Abbreviations: BSI, boundary shift integral; gBSI, generalized boundary shift integral.

**Fig. 7:**
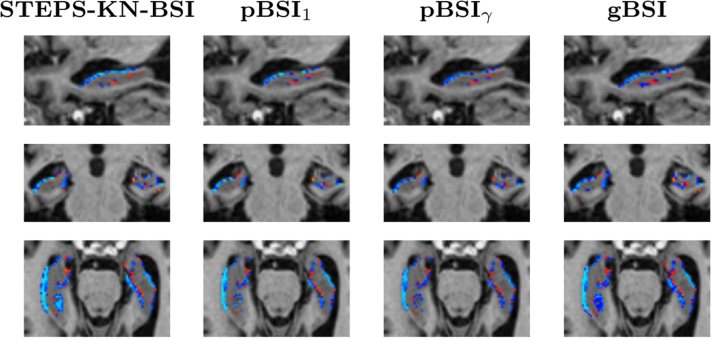
Example of atrophy voxelwise value for hippocampus area, for STEPS-KN-BSI, pBSI_1_, pBSI_*γ*_, and gBSI, obtained on an AD patient. Measured atrophy is represented in blue and/or light-blue and growth in red and/or orange. Abbreviations: AD, Alzheimer's disease; BSI, boundary shift integral; gBSI, generalized boundary shift integral. (For interpretation of the references to color in this Figure, the reader is referred to the web version of this article.)

For evaluation, we compared gBSI, pBSI, and KN-BSI using manual segmentations (referred to as “manual-KN-BSI”) and binarised segmentations from STEPS (referred to as “STEPS-KN-BSI”) (see Figs. 6 and 7). To investigate the effect of gBSI on both large and small structures, we applied the methods to calculate the whole brain and hippocampal atrophy rates. However, as manual hippocampal regions were not available for most subjects, the manual-KN-BSI method was excluded from the hippocampal analysis.

A double intensity-window KN-BSI was used to calculate the hippocampal atrophy rates (Leung et al., 2010a). The BSI double intensity window approach was previously described in Hobbs et al. (2009). The double intensity window was included to capture boundary shift at both the hippocampus-CSF border and the hippocampus-WM border. The optimal intensity window parameters were chosen using the same automatic intensity window selection method used by the single window approach.

As STEPS segmentations are not identical to manual segmentations, we included STEPS-KN-BSI in the comparison to understand if the improvement in gBSI comes from the probabilistic formulation or from the binarised STEPS segmentations.

To show the difference between pBSI and gBSI XOR mask, we have used 2 versions of pBSI XOR mask in a KN-BSI pipeline, that improves previous classic-BSI method (Freeborough and Fox, 1997) used in Ledig et al. (2012), performing a tissue-specific intensity normalization and automated intensity window selection. The 2 versions of pBSI XOR are obtained using the proposed parameters in Ledig et al. (2012) (*η* = 0.95, *n*_*e*_ = 0, *ζ* = 0.90, and *n*_*d*_ = 1), one is referred as pBSI_1_ and used as weighting function *γ* ≡ 1, and the other is pBSI_*γ*_ that corresponds to *γ* ≡ 0.5.

As there are no ground truths available for atrophy measures, we attempted to validate the algorithms by evaluating group separation in the context of measuring disease modification in a hypothetical clinical trial for AD. We compared the sample sizes required from manual-KN-BSI, STEPS-KN-BSI, pBSI_1_, pBSI_*γ*_, and gBSI. Annualized Percentage Brain Volume Change (PBVC) was calculated by dividing the BSI value by the volume of binarised baseline registered mask and the scan interval. Sample sizes per arm in a hypothetical AD trial (80% power at the 5% significance level) to detect 25% reduction in disease progression both with and without controlling for normal aging in controls were calculated using the following formula:

(7) $$\text{Sample size}=\frac{{\left(0.841+1.96\right)}^{2}\left(2\sigma^{2}\right)}{\Delta^{2}}$$

where *σ* denotes the variance in the treatment and placebo groups (assuming *σ* is the same in treatment and placebo groups (Fox et al., 2000)). Δ is the change in annualized PBVC between the treatment groups.

We obtained bias-corrected bootstrap CIs (10,000 bootstrap samples) for each of the estimated sample sizes and also for the ratio of the sample sizes between different methods. Statistical analyses were performed using Stata version 10 (College Station, TX, USA).

## 4 Results

Table 2 shows the mean and standard deviation of the annualized PBVC. The mean PBVC in controls were quite similar in manual-KN-BSI, STEPS-KN-BSI, and gBSI for 1.5 T and 3 T scans, although some of these differences, although less than 0.1% absolute atrophy, were significant using a paired *t* test. When comparing gBSI with pBSI in the same subjects, the pBSI algorithm results in significantly lower atrophy values in both controls and AD for 1.5 T and 3 T. Significantly lower atrophy was most notable in the pBSI_*γ*_ for 1.5 T and 3 T scans.

Table 3 shows the estimated sample sizes calculated from manual-KN-BSI, STEPS-KN-BSI, and gBSI. Similar sample sizes were obtained for manual-KN-BSI and STEPS-KN-BSI (*p* > 0.05). We obtained a smaller sample size for gBSI using 1.5 T scans, with a 13% (211–183) reduction when compared with manual-KN-BSI and a 10% (203–183) reduction when compared with STEPS-KN-BSI. Reductions of 6% when controlling for aging are also present when comparing gBSI with pBSI_*γ*_. We did not detect any statistical difference in sample size between different methods in 3T, possibly because of the smaller number of available 3T scans compared with 1.5 T scans.

Tables 4 and 5 show the results for the hippocampal experiments. Although the atrophy rates in gBSI are significantly lower than the same measures from STEPS-KN-BSI, there are no differences in the resulting sample size. However, the pBSI_1_ values result in significantly lower atrophy rates for 1.5 T scans and significantly higher sample sizes required for 1.5 T scans. Although pBSI_*γ*_ values result in significantly higher sample sizes required for both groups.

## 5 Conclusions and discussion

This work presents a generalized BSI method for measuring brain atrophy rates, and we show that it produces a modest but significant reduction in sample sizes needed in clinical trials in comparison with binarised implementation of the BSI. The generalization of BSI is obtained using a multi-atlas propagation and label fusion segmentation algorithm (Cardoso et al., 2013) with an adaptive selection of a spatial window. The pXOR region is adaptively adjusted according to the boundary uncertainty, improving boundary delineation. The smaller sample sizes obtained using gBSI suggests an increased sensitivity to change through the use of the probabilistic XOR region.

We also obtained smaller sample size estimation using KN-BSI in our proposed pipeline when compared with a previous pipeline in Leung et al. (2010b). This may be because of the use of extra N3 inhomogeneity correction step and other factors in preprocessing steps, for example, the degrees of freedoms used in the registration (Leung et al., 2012). We plan to fully investigate the cause of the improvement in future. It is worth noting that these sample sizes are equivalent despite gBSI showing lower atrophy rates. As seen in Figs. 4 and 5, the XOR region for both KN-BSI and gBSI span the same extent of the boundary, but the gBSI has lower weight around the edges reducing any atrophy observed in these areas. This also likely reduces the effect of non-brain or non-hippocampal tissue contributing to the atrophy measurement, thus also the reduced standard deviations.

We have obtained similar sample sizes using manual-KN-BSI and STEPS-KN-BSI, which provides further validation of the use of STEPS segmentations in BSI. For prevention or predementia trials at risk of developing AD (presymptomatic mutation carriers or asymptomatic patients at risk for sporadic AD due to signs of amyloid deposition or carrying an APOE ε4 allele), likely thousands of subjects will be required to observe decreased atrophy rates with suitable statistical power. As a result, the implementation of a reliable automatic segmentation into the atrophy pipeline will be needed to avoid a large group of manual rates that would be needed to meet the processing demands of the study.

The difference in sample size rates for STEPS-KN-BSI and gBSI are significant for the brain area but not for the hippocampal area. This shows that gBSI is more sensitive for detecting volume change in structures thanks to the adaptively weighted probabilistic spatial information incorporated in the XOR region (Manjón et al., 2010).

The presented pipeline is fully automated. STEPS remove the manual intervention in the segmentation step. Probabilistic XOR region reduces significantly the number of setting parameters needed. We have obtained a repeatable, reproducible, and standardized image-analysis pipeline, that it is the most desirable for clinical trials (Schuster, 2007).

The proposed work is most similar to a previous BSI method using probabilistic brain segmentations (Ledig et al., 2012), where a boundary shift region using the dilated union region and the eroded intersection region, using fuzzy logic and parameterized thresholds. A posteriori, they apply a spatial information function for getting the final probabilistic mask, used to compute BSI, computing it as the maximum between 0.5 and the probability segmentation mask of each time point (Ledig et al., 2012). Instead, we used probabilistic XOR adaptively weighted using the factor *κ* without using binary dilation and erosion and obtained directly from the combination of both probability mask. gBSI appears to provide lower sample sizes than the pBSI when using one of the suggested weighting functions, *γ* ≡ 0.5. When examining the pBSI regions in Fig. 5, we observe that they do not cover the same extent as the KN-BSI or gBSI, likely the cause for underestimating some of the regions. That the fuzzy implementation proposed by pBSI underestimates atrophy rates compared with KN-BSI is consistent with results presented by Ledig et al. (2012).

However, there are important differences in the intensity manipulation between pBSI and gBSI pipelines that directly affect the results. BSI is a biomarker that needs consistent image quality between time points to avoid possible bias. pBSI used a fixed single intensity window for measuring atrophy in hippocampus, without correcting the residual intensity inhomogeneity-derived differences between the baseline and the repeat images. For fixing this issue in our pipelines, we applied symmetric differential bias correction (Lewis and Fox, 2004). Moreover, we took into account that different images acquired from different sites may have different tissue contrasts and signal-to-noise ratios, and this means that each one has its optimal BSI intensity window. Ideally, its choice should be automated and unbiased (Leung et al., 2010b). Finally, using a single intensity window pBSI mainly captured the atrophy produced in the CSF-hippocampal border, ignoring most of the white matter-hippocampal border. To account for atrophy at this border, we applied a double intensity window (Hobbs et al., 2009; Leung et al., 2010a). These are the source of the difference between Ledig et al. (2012) and presented atrophy rates and sample sizes in hippocampus.

Several studies have previously compared BSI with related methods. Smith et al. (2007) obtained that SIENA and BSI has a good correlation and established that SIENA gives 20% larger atrophy values. Later studies found a consistent overestimation of SIENA with respect to BSI: 29% times in Camara et al. (2008) or 115% times in Sharma et al. (2010). Recently, Duran-Dubief et al. (2012) assessed the robustness of 7 different segmentation-based atrophy pipelines over multiple sclerosis patients. Holland et al. (2012) did an unbiased comparison of sample size estimates between different techniques (QUARC, TBM, FS, and BSI), where the best whole brain bias-corrected sample size estimation for AD value was obtained by KN-BSI, 75 (CI, 58–104), which is improved by gBSI in this work.

There are some potential caveats that need to be taken into account in this article. BSI, like the most modern atrophy biomarkers, is already quite robust, and most improvements will not have a dramatic effect. As there was less benefit on smaller regions like the hippocampus, further work will also assess the influence of both the gain factor *κ* and the intensity normalization strategy in the atrophy estimates and how these might be optimized depending on the region of interest.

## Disclosure statement

Professor Fox has an NIHR Senior Investigator award and receives support from the Wolfson Foundation; NIHR Biomedical Research Unit (Dementia) at UCL; the Engineering and Physical Sciences Research Council; Alzheimer's Research UK; and the National Institute on Aging. Professor Fox receives no personal compensation for the activities mentioned previously. Other authors report no disclosures.

NCF's research group has received payment for consultancy or for conducting studies from AVID, Bristol-Myers Squibb Foundation, Elan, Eisai, Lilly Research Laboratories, GE Healthcare, IXICO, Janssen Alzheimer Immunotherapy, Johnson & Johnson, Janssen-Cilig, Lundbeck, Neurochem Inc, Novartis Pharma AG, Pfizer Sanofi-Aventis and Wyeth Pharmaceuticals. Other authors' institutions have not contracts or other financial agreements or interests related to this work. The financial support of this work is disclosed in the Acknowledgements section of the manuscript. The data submitted as part of the current analyses have not been submitted elsewhere and will not be submitted elsewhere while under consideration at Neurobiology of Aging. All authors have reviewed the contents of the manuscript and approved its contents and validate the accuracy.

## Figures and Tables

**Table 1:** Full parameter selection used by gBSI

Processing step	Parameter name	Value	Reference
N3	Spline distance	150	Boyes et al. (2008)
	FWHM	0.05	
	Stopping threshold	0.0001	
	Iterations	1000	
	Resampling value	2	
Segmentation	Top brain templates to be fused	30	Cardoso et al. (2013)
	Top hippocampus templates to be fused	15	
Registration	Mask dilations	8	Leung et al. (2012)
DBC	Kernel size	5	Lewis and Fox (2004)
K-means	Mask dilations	3	Leung et al. (2010a)

Key: DBC, differential bias correction; gBSI, generalized boundary shift integral.

**Table 2:** Mean (SD) of annualized whole-brain PBVC atrophy rates between manual-KN-BSI, STEPS-KN-BSI, pBSI_1_, pBSI_*γ*_, and gBSI for ADNI

	Manual-KN-BSI	STEPS-KN-BSI	pBSI_1_	pBSI_*γ*_	gBSI
1.5 T					
Controls (N = 195)	0.56 (0.60)	0.55 (0.56)	0.49 (0.51)	0.34 (0.36)	0.53 (0.56)
AD (N = 133)	1.40 (0.77)	1.35 (0.72)	1.25 (0.65)	0.88 (0.48)	1.34 (0.69)
3 T					
Controls (N = 39)	0.45 (0.79)	0.40 (0.73)	0.39 (0.75)	0.22 (0.51)	0.39 (0.71)
AD (N = 24)	1.26 (0.71)	1.22 (0.70)	1.24 (0.72)	0.81 (0.47)	1.20 (0.69)

Comparison	Difference in mean (95% CI), *p*-value	
	1.5 T	3T
Manual-KN-BSI versus STEPS-KN-BSI		
Controls	0.008 (−0.009 to 0.025), 0.3	0.050 (−0.006 to 0.107), <0.1
AD	0.050 (0.024–0.076), <0.001	0.036 (−0.016 to 0.089), 0.16
Manual-KN-BSI versus gBSI		
Controls	0.029 (0.011–0.047), <0.01	0.059 (0.001–0.117), <0.05
AD	0.060 (0.033–0.088), <0.001	0.059 (0.005–0.113), <0.05
STEPS-KN-BSI versus gBSI		
Controls	0.021 (0.005–0.037), <0.01	0.008 (−0.002 to 0.019), 0.1
AD	0.010 (−0.005 to 0.025), 0.2	0.022 (0.011–0.033), <0.001
pBSI1 versus gBSI		
Controls	−0.042 (−0.052 to −0.031), <0.001	0.001 (−0.021 to 0.021), 0.99
AD	−0.091 (−0.103 to −0.078), <0.001	0.031 (0.012–0.050), <0.005
pBSIγ versus gBSI		
Controls	−0.191 (−0.221 to −0.161), <0.001	−0.172 (−0.248 to −0.096), <0.001
AD	−0.454 (−0.493 to −0.415), <0.001	−0.398 (−0.499 to −0.297), <0.001

Key: AD, Alzheimer's disease; ADNI, Alzheimer's Disease Neuroimaging Initiative; BSI, boundary shift integral; CI, confidence interval; gBSI, generalized boundary shift integral; PBVC, percentage brain volume change; SD, standard deviation.

**Table 3:** Estimated sample sizes (95% CI) per arm using whole brain annualized PBVC (80% power at the 5% significance level to detect 25% reduction in disease progression) with and without controlling for normal aging calculated from manual-KN-BSI, STEPS-KN-BSI, pBSI_1_, pBSI_*γ*_, and gBSI for ADNI

	Manual-KN-BSI	STEPS-KN-BSI	pBSI_1_	pBSI_*γ*_	gBSI
1.5 T					
Based on AD atrophy rates alone (N = 133)	76 (58–100)	71 (53–96)	68 (51–93)	75 (56–99)	66 (50–90)
Controlling for normal aging (N = 328)	211 (142–340)	203 (137–327)	185 (125–285)	197 (135–301)	183 (123–284)
3 T					
Based on AD atrophy rates alone (N = 24)	81 (37–162)	82 (40–158)	84 (40–162)	86 (41–171)	82 (40–159)
Controlling for normal aging (N = 63)	194 (70–854)	179 (72–675)	180 (70–644)	161 (61–573)	179 (69–629)

Comparison	Percentage difference of sample size (95% CI), *p*-value	
	1.5 T	3T
Manual-KN-BSI versus STEPS-KN-BSI		
AD rates alone	−6.6 (−13.4 to 0.6), >0.05	1.4 (−10.9 to 20.1), >0.05
Controlling for aging	−3.8 (−13.2 to 6.4), >0.05	−0.8 (−29.1 to 15.9), >0.05
Manual-KN-BSI versus gBSI		
AD rates alone	−12.1 (−18.8 to −5.0), <0.05	−1.7 (−10.8 to 20.6), >0.05
Controlling for aging	−13.3 (−21.6 to −4.2), <0.05	−7.5 (−28.9 to 16.6), >0.05
STEPS-KN-BSI versus gBSI		
AD rates alone	−6.5 (−10.6 to −2.3), <0.05	−0.3 (−3.3 to 3.3), >0.05
Controlling for aging	−9.8 (−16.0 to −3.5), <0.05	0.0 (−4.8 to 4.3), >0.05
pBSI1 versus gBSI		
AD rates alone	−2.4 (−5.0 to 0.6), >0.05	−3.2 (−6.6 to 1.2), >0.05
Controlling for aging	−1.0 (−4.4 to 3.0), >0.05	−0.6 (−6.5 to 5.2), >0.05
pBSIγ versus gBSI		
AD rates alone	−10.7 (−15.2 to −5.8), <0.05	−4.8 (−13.6 to 9.0), >0.05
Controlling for aging	−7.0 (−12.5 to −1.1), <0.05	1.1 (−6.0 to 35.2), >0.05

Key: AD, Alzheimer's disease; ADNI, Alzheimer's Disease Neuroimaging Initiative; BSI, boundary shift integral; CI, confidence interval; gBSI, generalized boundary shift integral; PBVC, percentage brain volume change.

**Table 4:** Mean (SD) of hippocampal PBVC atrophy rates among STEPS-KN-BSI, pBSI_1_, pBSI_*γ*_, and gBSI

	STEPS-KN-BSI	pBSI_1_	pBSI_*γ*_	gBSI
1.5 T				
Controls (N = 195)	1.12 (2.20)	0.69 (1.62)	0.40 (1.25)	0.87 (1.86)
AD (N = 133)	4.88 (3.23)	2.91 (2.23)	1.63 (1.72)	3.90 (2.54)
3 T				
Controls (N = 39)	0.35 (2.27)	0.37 (2.30)	0.21 (1.53)	0.15 (1.86)
AD (N = 24)	3.35 (3.08)	3.34 (3.1)	1.74 (2.35)	2.61 (2.46)

Comparison	Difference in mean (95% CI), *p*-value	
	1.5 T	3T
STEPS-KN-BSI versus gBSI		
Controls	0.249 (0.151–0.347), <0.001	0.207 (−0.378 to 0.452), <0.01
AD	0.988 (0.793–1.184), <0.001	0.737 (0.387–1.086), <0.001
pBSI1 versus gBSI		
Controls	−0.185 (−0.244 to −0.126), <0.001	0.228 (−0.026 to 0.482), <0.1
AD	−0.987 (−1.099 to −0.875), <0.001	0.724 (0.363–1.086), <0.001
pBSIγ versus gBSI		
Controls	−0.469 (−0.581 to −0.357), <0.001	−0.061 (−0.235 to 0.358), 0.679
AD	−2.264 (−2.473 to −2.055), <0.001	−0.874 (−1.282 to −0.465), <0.001

Key: AD, Alzheimer's disease; BSI, boundary shift integral; CI, confidence interval; gBSI, generalized boundary shift integral; PBVC, percentage brain volume change; SD, standard deviation.

**Table 5:** Estimated sample sizes (95% CI) per arm (80% power at the 5% significance level to detect 25% reduction in disease progression) with and without controlling for normal aging using the STEPS-KN-BSI, pBSI_1_, pBSI_*γ*_, and gBSI taking into account the hippocampal atrophy rates

	STEPS-KN-BSI	pBSI_1_	pBSI_*γ*_	gBSI
1.5 T				
Based on AD atrophy rates alone (N = 133)	109 (81–150)	147 (106–209)	281 (186–458)	106 (78–146)
Controlling for normal aging (N = 328)	184 (127–271)	253 (165–412)	495 (285–1002)	177 (121–271)
3T				
Based on AD atrophy rates alone (N = 24)	212 (83–700)	217 (83–769)	457 (153–3265)	222 (82–840)
Controlling for normal aging (N = 63)	265 (92–1320)	276 (96–1310)	590 (168–9900)	250 (83–1329)

Comparison	Percentage difference of sample size (95% CI)	
	1.5 T	3T
STEPS-KN-BSI versus gBSI		
AD rates alone	−2.7 (−13.4 to 9.7), >0.05	4.8 (−20.9 to 31.8), >0.05
Controlling for aging	−4.18 (−16.3 to 10.2), >0.05	−5.8 (−31.7 to 26.2), >0.05
pBSI1 versus gBSI		
AD rates alone	−27.8 (−36.5 to −19.4), <0.05	2.4 (−23.9 to 34.5), >0.05
Controlling for aging	−30.1 (−40.6 to −20.2), <0.05	−9.4 (−36.4 to 25.8), >0.05
pBSIγ versus gBSI		
AD rates alone	−62.0 (−72.7 to −51.7), <0.05	−51.3 (−76.3 to −20.3), <0.05
Controlling for aging	−64.2 (−76.8 to −51.1), <0.05	−57.7 (−86.5 to −22.6), <0.05

Key: AD, Alzheimer's disease; CI, confidence interval; gBSI, generalized boundary shift integral.

## References

[bib1] Anderson V.M., Fox N.C., Miller D.H. (2006). Magnetic resonance imaging measures of brain atrophy in multiple sclerosis. J. Magn. Reson. Imaging.

[bib2] Boyes R.G., Gunter J.L., Frost C., Janke A.L., Yeatman T., Hill D.L.G., Bernstein M.A., Thompson P.M., Weiner M.W., Schuff N., Alexander G.E., Killiany R.J., DeCarli C., Jack C.R., Fox N.C. (2008). Intensity non-uniformity correction using N3 on 3-T scanners with multichannel phased array coils. Neuroimage.

[bib3] Camara O., Schnabel J.A., Ridgway G.R., Crum W.R., Douiri A., Scahill R.I., Hill D.L.G., Fox N.C. (2008). Accuracy assessment of global and local atrophy measurement techniques with realistic simulated longitudinal Alzheimer’s disease images. Neuroimage.

[bib4] Cardoso M.J., Leung K., Modat M., Keihaninejad S., Cash D., Barnes J., Fox N.C., Ourselin S. (2013). STEPS: similarity and truth estimation for propagated segmentations and its application to hippocampal segmentation and brain parcelation. Med. Image Anal..

[bib5] Duran-Dubief F., Belaroussi B., Armspach J.-P., Roggerone S., Vukusic S., Hannoun S., Cotton F. (2012). Reliability of longitudinal brain volume loss measurements between 2 sites in patients with multiple sclerosis: comparison of 7 quantification techniques. AJNR Am. J. Neuroradiol..

[bib6] Fox N.C., Cousens S., Scahill R., Harvey R.J., Rossor M.N. (2000). Using serial registered brain magnetic resonance imaging to measure disease progression in Alzheimer disease. Arch. Neurol..

[bib7] Freeborough P.A., Fox N.C. (1997). The boundary shift integral: an accurate and robust measure of cerebral volume changes from registered repeat MRI. IEEE Trans. Med. Imaging.

[bib8] Frisoni G.B., Fox N.C., Jack C.R., Scheltens P., Thompson P.M. (2010). The clinical use of structural MRI in Alzheimer disease. Nat. Rev. Neurol..

[bib9] Grill J.D., Di L., Lu P.H., Lee C., Ringman J., Apostolova L.G., Chow N., Kohannim O., Cummings J.L., Thompson P.M., Elashoff D. (2013). Estimating sample sizes for predementia Alzheimer’s trials based on the Alzheimer’s Disease Neuroimaging Initiative. Neurobiol. Aging.

[bib10] Gunter J., Bernstein M., Borowski B., Felmlee J., Blezek D., Mallozzi R., Levy J., Schuff N., Jack C. (2006). Validation Testing of the MRI Calibration Phantom for the Alzheimer’s Disease Neuroimaging Initiative Study.

[bib11] Gustaw-Rothenberg K., Lerner A., Bonda D. (2010). Biomarkers in Alzheimer’s disease: past, present and future. Biomarkers Med..

[bib12] Hobbs N.Z., Henley S.M.D., Wild E.J., Leung K.K., Frost C., Barker R.A., Scahill R.I., Barnes J., Tabrizi S.J., Fox N.C. (2009). Automated quantification of caudate atrophy by local registration of serial MRI: evaluation and application in Huntington’s disease. Neuroimage.

[bib13] Holland D., Dale A.M. (2011). Nonlinear registration of longitudinal images and measurement of change in regions of interest. Med. image Anal..

[bib14] Holland D., McEvoy L.K., Dale A.M. (2012). Unbiased comparison of sample size estimates from longitudinal structural measures in ADNI. Hum. Brain Mapp..

[bib15] Hua X., Hibar D.P., Ching C.R.K., Boyle C.P., Rajagopalan P., Gutman B.A., Leow A.D., Toga A.W., Jack C.R., Harvey D., Weiner M.W., Thompson P.M. (2013). Unbiased tensor-based morphometry: improved robustness and sample size estimates for Alzheimer’s disease clinical trials. Neuroimage.

[bib16] Jovicich J., Czanner S., Greve D., Haley E., van der Kouwe A., Gollub R., Kennedy D., Schmitt F., Brown G., Macfall J., Fischl B., Dale A. (2006). Reliability in multi-site structural MRI studies: effects of gradient non-linearity correction on phantom and human data. Neuroimage.

[bib17] Ledig, C., Wolz, R., Aljabar, P., Lötjöven, J., Rueckert, D., 2012. PBSI: a symmetric probabilistic extension of the boundary shift integral. In: workshop on novel imaging biomarkers for Alzheimer’s disease and related disorders (MICCAI’12). p. 117–24.

[bib18] Leung K.K., Barnes J., Ridgway G.R., Bartlett J.W., Clarkson M.J., Macdonald K., Schuff N., Fox N.C., Ourselin S. (2010). Automated cross-sectional and longitudinal hippocampal volume measurement in mild cognitive impairment and Alzheimer’s disease. Neuroimage.

[bib19] Leung K.K., Clarkson M.J., Bartlett J.W., Clegg S.L., Jack C.R., Weiner M.W., Fox N.C., Ourselin S. (2010). Robust atrophy rate measurement in Alzheimer’s disease using multi-site serial MRI: tissue-specific intensity normalization and parameter selection. Neuroimage.

[bib20] Leung K.K., Ridgway G.R., Ourselin S., Fox N.C. (2012). Consistent multi-time-point brain atrophy estimation from the boundary shift integral. Neuroimage.

[bib21] Lewis E.B., Fox N.C. (2004). Correction of differential intensity inhomogeneity in longitudinal MR images. Neuroimage.

[bib22] Lindley D.V. (1987). The probability approach to the treatment of uncertainty in artificial intelligence and expert systems. Stat. Sci..

[bib23] Manjón J.V., Tohka J., Robles M. (2010). Improved estimates of partial volume coefficients from noisy brain MRI using spatial context. Neuroimage.

[bib24] Modat M., Cash D.M., Daga P., Winston G.P., Duncan J.S., Ourselin S., Martin A Styner (2014). A symmetric block-matching framework for global registration. SPIE Medical Imaging 2014.

[bib25] Narayana P., Brey W., Kulkarni M., Sievenpiper C. (1988). Compensation for surface coil sensitivity variation in magnetic resonance imaging. Magn. Reson. Imaging.

[bib26] Ourselin S., Roche A., Subsol G., Pennec X., Ayache N. (2001). Reconstructing a 3D structure from serial histological sections. Image Vis. Comput..

[bib27] Reuter M., Rosas H.D., Fischl B. (2010). Highly accurate inverse consistent registration: a robust approach. Neuroimage.

[bib28] Reuter M., Schmansky N.J., Rosas H.D., Fischl B. (2012). Within-subject template estimation for unbiased longitudinal image analysis. Neuroimage.

[bib29] Schott J.M., Bartlett J.W., Barnes J., Leung K.K., Ourselin S., Fox N.C. (2010). Reduced sample sizes for atrophy outcomes in Alzheimer’s disease trials: baseline adjustment. Neurobiol. Aging.

[bib30] Schuster D.P. (2007). The opportunities and challenges of developing imaging biomarkers to study lung function and disease. Am. J. Respir. Crit. Care Med..

[bib31] Sharma S., Noblet V., Rousseau F., Heitz F., Rumbach L., Armspach J.P. (2010). Evaluation of brain atrophy estimation algorithms using simulated ground-truth data. Med. image Anal..

[bib32] Sled J., Zijdenbos A., Evans A. (1998). A nonparametric method for automatic correction of intensity nonuniformity in MRI data. Med. Imaging IEEE Trans..

[bib33] Sluimer J.D., Bouwman F.H., Vrenken H., Blankenstein M.A., Barkhof F., van der Flier W.M., Scheltens P. (2010). Whole-brain atrophy rate and CSF biomarker levels in MCI and AD: a longitudinal study. Neurobiol. Aging.

[bib34] Smith S.M., De Stefano N., Jenkinson M., Matthews P.M. (2001). Normalized accurate measurement of longitudinal brain change. J. Comput. Assist. Tomogr..

[bib35] Smith S.M., Rao A., De Stefano N., Jenkinson M., Schott J.M., Matthews P.M., Fox N.C. (2007). Longitudinal and cross-sectional analysis of atrophy in Alzheimer’s disease: cross-validation of BSI, SIENA and SIENAX. Neuroimage.

[bib36] Smith S.M., Zhang Y., Jenkinson M., Chen J., Matthews P.M., Federico A., De Stefano N. (2002). Accurate, robust, and automated longitudinal and cross-sectional brain change analysis. Neuroimage.

[bib37] Wyman B.T., Harvey D.J., Crawford K., Bernstein M.A., Carmichael O., Cole P.E., Crane P.K., DeCarli C., Fox N.C., Gunter J.L., Hill D., Killiany R.J., Pachai C., Schwarz A.J., Schuff N., Senjem M.L., Suhy J., Thompson P.M., Weiner M., Jack C.R. (2013). Standardization of analysis sets for reporting results from ADNI MRI data. Alzheimer’s Dement..

